# circRNA-miRNA-mRNA regulatory network in human lung cancer: an update

**DOI:** 10.1186/s12935-020-01245-4

**Published:** 2020-05-19

**Authors:** Zhuo-Zheng Liang, Cheng Guo, Man-Man Zou, Ping Meng, Tian-Tuo Zhang

**Affiliations:** 1 Department of Pulmonary Diseases, The Third Affiliated Hospital of Sun Yat-sen University, Institute of Respiratory Diseases of Sun Yat-sen University, 600 Tianhe Road, Guangzhou, 510630 China; 2 Department of Otolaryngology-Head and Neck Surgery, The Third Affiliated Hospital of Sun Yat-sen University, Guangzhou, China

**Keywords:** Circular RNAs, Lung neoplasms, ceRNA-miRNA-mRNA, miRNA sponge

## Abstract

Circular RNAs, as hopeful diagnosis markers and therapeutic molecules, have been studied, probed and applied into several diseases, such as cardiovascular diseases, systemic lupus erythematosus, leukemia, pulmonary tuberculosis, and cancer especially. Recently, mounting evidence has supported that circRNAs play a key role in the tumorigenesis, progress, invasion and metastasis in lung cancer. Its special structure—3′–5′ covalent loop—allow it to execute several special functions in both normal eukaryotic cells and cancer cells. Our review summaries the latest studies on characteristics and biogenesis of circRNAs, and highlight the regulatory functions about miRNA sponge of lung-cancer-related circRNAs. In addition, the interaction of the circRNA-miRNA-mRNA regulatory network will also be elaborated in detail in this review. Therefore, this review can provide a new idea or strategy for further development and application in clinical setting in terms of early-diagnosis and better treatment.

## Background

In the 21st century, cancer is still one of the most life threating diseases of the global health and wasting ever-increasing amounts of medical resources at the global level, and lung cancer is a highly heterogeneous malignance disease, which reaches the first in both the incidence and mortality of all-cancer (accounting for 11.6% of the overall total of 18.1 million new cases and 18.4% of the total number of 9.6 million cancer deaths respectively) in 2018 [1]. Thus, future curative strategies should be made to gain better understanding of cancer development, early diagnosis and effective treatment in oncology. In recent years, drug precise treatments targeting proteins encoding by EGFR, ALK, KRAS, mTOR, ErbB2 and PI3K, and anti-tumor immunity immune check point blockade, like PD-1, PD-L1 and CTLA4 inhibitors, emerge as promising treatments for human malignancies especially a more profound antitumor effect in patients with advanced lung carcinoma [2, 3]. By in-depth study, development of the new molecules therapeutic targets, including non-coding RNAs (ncRNAs), are the novel subjects of the research program. microRNA (miRNA), long non-coding RNA (lncRNA) and circular RNA (circRNA) are the top three members of ncRNAs in researches when search in the Pubmed. Arguably, increasing accumulating researches have been carried out to explicate the functions of circRNAs. Considered as the noisy transcripts for a long time at the beginning, circRNAs are kinds of single-stranded circular transcripts with a 3′–5′ covalently closed link [4]. Thanks to this special structure and to the fact that, unlike normal linear mRNA, circRNAs keep stable and resist to exonuclease. Derived from exons and (or) introns, circRNAs have certain evolutionary conservation and feature as tissue-specific expression.

Although Ma et al. have summarized the characteristics, biogenesis, function and progress of circRNA in lung cancer up to May 2018 [5], many published studies report new circRNAs involved in diagnosis biomarkers and therapeutic molecules in 2018. The current review was aimed to update the knowledge of circRNAs in lung cancer and focusing more on the effect of circRNA-miRNA-mRNA regulatory axes in lung cancer.

## Characteristics of circRNA

CircRNAs, identified as brand-new star molecules, are abundantly expressed in a variety of eukaryotic cells, that are transcribed from a considerable number of genes both in human and animals [6]. Without the 5′-cap structure and 3′-ploy(A) tail, circRNAs with a structure of closed loop covalently links from 5′-head to 3′-tail [4]. In contrast with linear RNAs, a free 3′ overhang with at least 7 nucleotides required for exoribonuclease, circRNAs are more resistant to degradation by RNase or RNA exonucleases so that circRNAs are able to maintain a stable loop [7]. In 1970s, Sanger et al. confirmed the existence of circRNA firstly in viroid propagated in tomatoes and then 3 years later, circRNA was discovered in eukaryotic cells by electron microscope [8]. Due to the limitation of the technology, circRNAs are considered as nonsense RNAs or transcripts noises for a long time, which led to less attentions to its biological functions. Integrated with advanced technology of RNA sequencing (RNA-seq) and bioinformatics, it was supported that circRNAs exerted superior properties on diagnosis and treatment in clinical setting. It is reported that circRNAs are less abundant than their cognate linear transcripts. Some tissue-specific circRNAs, however, express more highly than their linear transcripts produced from the same gene, such as circTulp4, circRims2, circElf2, circphf21a, etc. in brain tissue [9, 10]. Four features of circRNA can be concluded below: (1) *abundance and variety*: Memczak and colleagues found that 1950 circRNA in HEK 293 cells, 1903 circRNA in mouse, 1028 circRNA in zebrafish and 724 circRNA in different stages of *C. elegans* through RNA-seq. High-throughput sequencing profiles revealed that circRNAs are widely expressed in genome transcriptional profiles of different types of normal and malignant human cells [6, 11]. Additionally, some circRNAs express tenfold as many as their associated linear mRNAs [12]; (2) *stability*: after being injected into fertilized eggs of Xenous laevis, the half-life of circRNAs are at least fivefold longer than linear message RNAs (mRNAs) with or without 5′-cap and 3′-poly(A) [13]; (3) *conservation*: circRNAs produced by mouse orthologous location of human genes are similar to the RNA products encoding by the same locus of human gene [12]; (4) *clear location*: most mature circRNAs are located in the cytoplasm, while some of circRNAs containing introns are usually retained in the nucleus [14]; (5) *specificity* as novel biomarkers, different circRNAs can be secreted into serum from different tissues and cells. There is much evidence to indicate that circRNAs with high specificity and sensitivity play potential roles on many human diseases with respect to early-diagnosis and effective therapy [15].

## Classification of circRNA

In most eukaryotic cells, split genes encode RNAs with exons interrupted by introns, which are called precursor mRNAs (pre-mRNAs). Instead of canonical splicing using by linear mRNAs, circRNA transcripts generate themselves with a reverse mechanism, in which 3′ upstream donor connects with 5′ downstream acceptor, called head-to-tail back-splicing [16]. According to their constituent parts and sources, circRNAs can be briefly classified into nine types:

1. *exonic circRNAs (ecircRNAs)* EIciRNAs, which account for the main body of circRNAs (more than three fourths of the whole), circulate from head to tail with only one or several exons in the cytoplasm. The later are called exon skipping.
2. *exonic-intronic circRNAs (EIciRNAs)* EIciRNAs consist of both exons and introns which retain from back-splicing.
3. *intronic circRNA (ciRNAs)* CiRNAs prominently locate in nucleus and are generated from pre-mRNAs by removing the exons and closely connecting the intron’s head to its tail.
4. *intergenic circRNAs* These circRNAs are comprised of sequences between two intronic fragments flanked by GT-AG splicing signals, which locate in at least 1 kb away from two genes at both sides.
5. *tRNA intronic circRNAs (tricRNAs)* This kind of circRNAs is a special type of intronic circRNAs derived from pre-tRNAs. Intron-containing pre-tRNAs are cut by endonuclease complex resulting in forming intron transcripts and mature tRNAs, thereby tricRNAs were produced.
6. *antisense circRNAs* The formation mechanism of this circRNA is the same as ecircRNA, but happening in the opposite strand rather than sense strand.
7. *overlapping circRNAs*
8. *circRNA rRNAs (circrRNAs)*
9. *intragenic circRNAs* This type of circRNA is similar to intergenic circRNA, but the cyclization sequence is less than 1 kb away from either side between two genes [12, 17–21].

## Biogenesis of circRNA

The formation of both linear RNA and circRNA are processed by splicing transcriptional sequence derived from pre-mRNA. However, the splicing mechanisms of generating themselves are totally different. Pre-mRNA splicing requires the involvement of spliceosome. As for the formation mechanism of linear mRNAs, pre-mRNAs are processed by spliceosomal machinery, where introns can be removed and exons can be selectively linked in juxtaposition, subsequently resulting in generation of 5′–3′ linked linear transcripts through constitutive splicing (CS) and alternative splicing (AS) mechanism [22]. However, back-splicing in reserve order of canonical splicing, which connects the 3′ upstream splice site (3′ ss) to the 5′ downstream splice site (5′ ss), is the main producing mechanism for circRNA. Indeed, it is well known that three splicing models can be found in the biogenesis of the circRNA: (1) circulation driven by lariat; (2) circulation driven by intron-pairing; (3) splicing intermediated by RNA binding protein (RBP). The detail information about these mechanisms can be concretely described as follow in terms of biogenesis of circRNA. Taking ecircRNAs, EIciRNAs and ciRNAs for representative examples.

### EcircRNAs

Diverse ecircRNAs containing one or more exons are generally produced from splicing procedure of pre-mRNAs by the means of two models and three splicing mechanisms [23]. We can remember these as ‘3 + 2’. First, two models, namely, direct back-splicing and exon-skipping, can generate ecircRNAs with single one or several exons [4]. The main difference between two models is which pattern of splicing, canonical splicing or back-splicing, come first. Second, Lariat-driven cyclization, RBP-mediated cyclization and intron-pairing cyclization are the three mechanisms, which purpose to forming diverse ecircRNAs. Lariat-driven cyclization is the main mechanism in the splicing process of pre-mRNAs in the exon-skipping model, while RBP-mediated and intron-pairing cyclization are primarily responsible for the direct back-splicing [24]. For one thing, in the lariat-driven cyclization process, canonical splicing comes first, which will narrow the distance between upstream splice site and downstream splice site. Consequently, a linear mRNA with exons selective inclusion and introns exclusion, as well as a loop structure with one or more exons sequence flanking introns will be generated in the first step. And then spliceosome will cut the redundant introns and join 3′ hydroxyl of exon to 5′ phosphate, thus resulting in forming a 3′–5′ linked lariat-ecircRNA. Such process is called exon-skipping. For another thing, if trans- factors, like RBPs, bind to a pre-mRNA and cis-elements pairing brings the introns together, back-splicing will come first. Either RBPs or intron-pairing will dramatically promote back-splicing. Accordingly, ecircRNAs can be straightly generated at the very start [25]. Although the fact that back-splicing is much lower efficient than canonical splicing maybe attribute to the hypothesis that spliceosome unfavorably bind to pre-mRNA, RBP or introns-pairing can facilitate the splice process, complementing the disadvantage of efficiency of back-splicing [16, 26]. In addition, Memczak et al. identified that AG/GT flanking exons is a splice signal which plays a vital role for selecting breakpoint by spliceosome [6].

### EIciRNAs

Introns-containing of EIciRNAs is the major distinction from ecircRNAs with respect to constituent parts. Notably, the mechanisms of generating EIciRNAs are fundamentally similar to ecircRNAs’ [24]. During the formation of ecircRNA, an intermediate production including exons flanking unspliced introns will be generated, which can be further processed into ecircRNA or EIciRNA by splicing or retaining introns. In RBP-binding cyclization, RBPs, such as Muscleblind (MBL/MBNL1), Quaking (QKI), Fused-in sarcoma (FUS), NF90/NF110, DHX9, HNRNRL, et al. have been reported to contribute a lot in the biogenesis of circRNA, through binding to pre-mRNA, getting the introns together and promoting the RNA splicing enzymes binding to RNA–protein complex [27–31]. MBL protein specifically binds to conserved muscleblind binding sites of pre-mRNA encoding by mbl gene and promotes the spliceosome to strongly bind to pre-mRNA, resulting in upregulating the production of circMbl in Drosophila. Furthermore, functional MBL binding sites in the introns of pre-mRNA encoding by mbl gene are required for the increase production of circMbl [32]. In contrast, nuclear RNA helicase DHX9 acting as an inhibitor regulatory factor interacts with inverted complementary sequence and downregulate the splice process of circRNA. Recently, research results of Yu show that unregulated DHX9 contributing to downregulation of cSMARCA5 mediates the promotion of growth, aggression and metastasis of hepatocellular carcinoma (HCC) [33].

Additionally, as cis elements, intron-pairing can also increase the rate of producing circRNAs. Exons flanking introns with complementary reserved sequences will form like a loop by intron pairing. This structure is expected to bring the distal splice site to proximity, which is an efficient way to facilitate the back-splicing of circRNA [16]. One of the best known are ALU repetitive elements, which occupy approximately 10% of the whole genome [34]. Interestingly, short inverted sequences (30–40 nucleotides) like ALU repeats can dramatically enhance the production of circRNA, while longer repeats can sometimes inhibit the back splicing of circRNA [35]. It is reported that competition of RNA pairing exists between ALU repeats within different introns [26, 34]. Besides circRNA, ALU repeats also involve in the biogenesis of miRNA. Di Ruocco et al. found that accumulation of ALU sequences is associated with both epithelial-to-mesenchymal transition (EMT) and cancer progression by targeting miR-566, which can lead to upregulation of DICER1 [36]. Surprisingly, Zhang et al. observed that not only the short repetitive sequences can promote the splicing process of circRNA, but also the non-repeated elements contribute to pre-mRNA cyclization [16].

### CiRNAs

CiRNAs are identified as a sequence containing only introns and mainly perform their functions in the nucleus. Their sizes range from 200 to 3000 nucleotides [37]. It is generally believed that excised introns or removed remain introns will be easily and immediately degraded by RNase [38]. To generate a stable ciRNA after excised lariat introns are removed from spliceosome, some specific structures are usually necessary for during the splicing [39]. Zhang et al. reported that the reasons why ciRNAs fail to be debranched by enzymes are the existence of an special consensus motif that 7 nucleotides GU-rich sequences close to 5′ upstream splice site and 11 nucleotides C-rich sequence locate near in branch point of 3′ downstream splice site [40]. Treated by RNase on branch point of intron lariat, ciRNA is finally formed. Therefore, these elements play an important role in generating a stable ciRNA in the back splicing.

## Functions of circRNAs in cancer

Giving that circRNAs possess some special structures which can interact with DNAs, RNAs and even proteins, circRNAs have potential to be new multifunctional small molecules. Regulatory networks can be established when circRNAs bind to other molecules forming circRNA-DNA, circRNA-RNA, circRNA-protein interactions. Recent years have witnessed a flood of researches which focus on the functions and mechanisms of how circRNAs work biologically. Multiple functions of circRNAs are demonstrated in Table 1. Detail information will be classified into three parts as follow.

**Table 1:** Functions of circRNAs

Function	Example	References
miRNA sponge	circ-HIPK3 circ-PRKCI	[41, 42]
Translation regulator	circ-PABPN1	[43]
Alternative splicing	circ-Mbl	[44]
RNAP II elongation	circ-EIF3J circ-PAIP2	[18]
RNA maturation	circ-ANRIL	[45]
Protein translation (including m6A-driven)	circ-ZNF609	[46, 47]
Protein sponge	circ-Foxo3	[48]
Histone methylation	Circ-ANRIL	[49]

*Transcription* EIciRNAs such as circEIF3J or circPAIP2 naturally interact with U1 snRNP which is indispensable for the function of EIciRNAs where occur predominately in nucleus. EIciRNAs associate with RNA polymerase II and play a special role in promoting parental gene expression [18].

*Translation* Although circRNAs are identified as non-coding RNAs which canonically do not translate into proteins, some researchers found that circRNAs process a special function that can express translationally. Legnini et al. show that circ-ZNF609 contains a 753-nt open reading frame (ORF) and encode proteins in myogenesis [46]. Moreover, some initiation of circRNAs translation is driven by N6-methyladenosine (m6A). Yang et al. identified that circRNAs containing m6A motif can produce proteins and its translation efficiency can be control by regulation of m6A level. In-deep study shows that m6A motif recruit IF4G2 and YTHDF3 which requires for the initiation of translation [47]. Additionally, Abdelmohsen et al. identified that HuR proteins are able to bind to PABPN1 mRNA promoting mRNA translation. Targeting HuR, overexpressing circPABPN1 can be observed to upregulated PABPN1 abundance by keeping HuR from binding to its cognate transcript, eventually leading to low expression of PABPN1 [43].

*Others* Holdt found that in vascular smooth muscle cells and macrophages of human atherosclerosis, circANRIL is a member of a pre-ribosomal assembly complex by binding to pescadillo homologue 1 (PES1), which mediates the biogenesis and maturation of ribosome. This process leads to cell apoptosis and inhibits cell proliferation, which is independent on miRNA sponge [45]. CircRNAs not only can function as miRNA sponge, but also are able to sponge and interact with proteins. Du et al. reported that expression level of circFoxo3 in heart tissues of aging patients is higher than that of young patients. Of interest, experimentation using validated the fact that circFoxo3 interact with ID-1, E2F1, FAK and HIF-1α which increase cellular senescence by suppressing the effect of these proteins [48].

Most importantly, miRNA sponge will be described in detail below.

## Function of miRNA sponge in human cancer

circRNAs identified as competing endogenous RNAs (ceRNAs) are the parts of small non-coding RNAs which play big parts in post-transcription and participate in genetic expression transcriptionally and translationally. It is well known that a part of circRNAs containing miRNA response elements (MREs) can act as competing molecules by interacting with diverse miRNAs and affect the efficiency of miRNAs to regulate the downstream mRNA expression [50].

circRNAs have been studied in many human diseases, such as neurodegenerative diseases, neural injuries, cardiac hypertrophy, myocardial infarction, atherosclerosis and diverse malignant carcinomas [51]. The prevention, diagnosis and therapy of cancers are the hot topics for human in a long time. There is no hesitation that the cure for cancer is the unremitting struggle of human. circRNAs as the star molecules in diagnosis and therapy have been investigated in a great deal of malignant tumors. Taking ciRS-7 for examples. Li et al. found that ciRS-7 upregulates in esophageal squamous cell carcinoma (ESCC) while miR-7 downregulates in the same cancer tissues compared with para-carcinoma tissues. Furthermore, they verified that elevated ciRS-7 can eliminate the effect of inhibitory miR-7 which inhibits the proliferation and metastasis of ESCC. Knockdown of HOXB13 in elevated ciRS-7/miR-7 cells can negatively affect colony formation and migration of cancer cells [52]. For breast cancer, ciRS-7 can also contribute to its tumorigenesis and metastasis. The expression level of ciRS-7 in triple negative breast cancer (TNBC) is higher than other types breast cancer tissues from who patients did not undergo chemotherapy and radiotherapy before surgery. Knockdown of ciRS-7 can suppress the metastasis of TNBC in vivo and in vitro. Mechanistically, ciR-7 acting as miR-1299 sponge mediates the EMT by upregulating matrix metalloproteinases (MMPs) [53].

With the extensive and in-depth research on cancer in the past decades, tumor microenvironment (TME) has been founded and researched. TME refers to the outcome of interaction among various tumor cell types, and act as an important component in fields of tumor cells progressing, growing and metastasizing [54]. Surprisingly, numerous studies have demonstrated that circRNAs can affect TME via circRNA-miRNA-mRNA axis [55]. To this end, the circRNA-miRNA-mRNA axis on TME has been regarded as a promising breakthrough in possible preventive strategies, molecular diagnosis, and therapy strategies on cancer.

1. Regulate tumor immunosuppression
  During the past decades of TME research, programmed death 1/programmed death-ligand 1 (PD-1/PD-L1) has been identified as a crucial element to tumor immunosuppression [56]. Current research shows that due to the regulation of upstream circRNAs, the expression of PD-L1 can be affected by the corresponding circRNA-miRNA-PD-L1 axis. Subsequently, the interaction between PD-L1 and PD-1 can properly suppress the stimulator of effector T lymphocytes, eventually causing tumor cells to escape immune surveillance [57, 58]. The considerable roles of miRNAs in modulating PD-1/PD-L1 have been well investigated in the past few decades. However, the definite mechanism of circRNA regulates PD-1/PD-L1 through ceRNA is still unclear. One recent study revealed that circular RNA circ-0020397 was upregulated in colorectal cancer, circ-0020397 through circ-0020397 ↑-miR-138- ↓ PD-L1 ↑ to bind PD-1, inhibit T cell proliferation and activation, and induce T cell apoptosis, ultimately resulting in tumor immune escape [57]. As a consequence, the corresponding circRNAs through the ceRNA pathway to target and modulate PD-1/PD-L1 may become a novelty research direction for tumor immune escape and new treatment for cancer.
2. The cytotoxicity of natural killer cells
  Natural killer (NK) cells, known as a vital component defense for human immune surveillance. Increasing experimental evidence has indicated that once the NK cells recognize and destroy the target cells since there is no major histocompatibility complex to prevent it, more and more immune activators can be generated to enhance the destruction of the target cells [59]. Previous studies elucidated the underlying mechanism by that circRNAs’ involve in NK-mediated immune responses by the ceRNA pathway. For instance, in pancreatic cancer cells under hypoxia, circ-0000977 can pass circ-0000977-miR-153-Hypoxia-inducible factor 1-α (HIF1α) axis to regulate HIF1α -mediated immune escape [60].
3. Angiogenesis
  Tumor angiogenesis is an extremely complex process in biology [61]. Cancer cells can induce angiogenesis by affecting TME, thereby providing tumor cells with the oxygen and nutrients they need to pass through and provide a guarantee for tumor proliferation and invasion [60]. In recent decades, circRNAs have been reported to regulating tumor angiogenesis by playing as related downstream miRNA “sponges”. For example, a fascinating feedback loop namely FUS-circ-002136-miR-138-5p-SOX13 was discovered in glioma associated endothelial cells (GECs), and stimulating GECs angiogenesis [62]. Emerging research showed the expression of AGGF1 was increased by circSHKBP1-miR-379-FOXP2 or circSHKBP1-miR-544a/FOXP1 axis, which promoted the angiogenesis of glioma [63]. The above studies expound that circRNAs are important in regulating tumor angiogenesis through the mechanism of sponge miRNA. Therefore, we reasonably speculate that the ceRNA pathway of related circRNAs is a promising direction in the future research of tumor angiogenesis inhibitors.
4. Hypoxia regulates circRNA production
  In TME, hypoxia can cause a series of complex biological changes, therefore affecting the occurrence and progression of cancer. HIFs, a type of transcriptional regulator, plays a crucial role in regulating TME responses and tumor cell proliferation and metastasis by stimulating downstream oncogene transcription of hypoxic response elements and regulating various relevant signaling pathways [64]. Mounting evidence demonstrates that hypoxic TME-related circRNAs involve in metastasis, invasion, angiogenesis, and radiation-resistance [65]. For example, the hypoxia-induced expression of circDENND4C and the circDENND4C/miR-200b, miR-200c axis could effectively modulate glycolysis, migration and invasion in breast cancer cells [66]. Interestingly, another study illustrated that circDENND2A was induced by hypoxia in glioma cells, and the circDENND2A-miR-625-5p axis promotes invasion and migration in glioma cells via the miR-625-5p interacted with HIF1α [67]. For this reason, the ceRNA mechanism of circRNA plays an important role in the case of tumor hypoxia, which may become the focus of our future research.

In short, researches about circRNAs can be found in various cancers, such as breast cancer, hepatocellular carcinoma, lung cancer, gastric cancer, colon cancer, neuro glioma, et al. [68]. In my review, we mainly focus on the effect of circRNAs in lung cancer. The contribution of circRNA-miRNA-mRNA regulatory network to lung cancer will be highlighted as follow.

## circRNA-miRNA-mRNA regulatory axis in lung cancer

In order to fully understand the circRNAs on lung cancer, our team searched the related literatures on Pubmed, Embase and Web of Science. 53 literatures demonstrating the relationship or the application between circRNAs and lung cancer come into notice. However, taking into consideration that we shed light on circRNA-miRNA-mRNA regulatory network on lung cancer, only 43 individual researches can be included in this review (shown Table 2).

**Table 2:** The summary of validated circRNA-miRNA-mRNA regulatory axes in lung cancer

Cancer types	Sample	circRNA-miRNA-mRNA	Cell characteristics	Clinical characteristics	Clinical application	References
LAC	Tissues Plasma Cells		1. Cell proliferation 2. Cell invasion 3. Cell apoptosis 4. Cell migration	1. TNM stage 2. Lymph node metastasis	Diagnostic values AUC: 0.815 Sensitivity: 0.755 Specificity: 0.796	[69, 70]
NSCLC	Cells		1. Cell proliferation	–	–	[41]
NSCLC	Tissues Cells		1. Cell proliferation 2. Cell invasion 3. Cell apoptosis 4. Cell migration	1. T stage 2. Lymph node metastases 3. TNM stage 4. Poor differentiation	–	[71]
NSCLC	Tissues Cells		1. Cell proliferation 2. Cell apoptosis	1. TNM stage 2. Lymph node metastasis 3. Survival rate	Prognostic value	[72]
NSCLC	Tissues Cells		1. Cell proliferation 2. Cell invasion 3. Cell apoptosis 4. Cell migration	1. TNM stage 2. Lymph node metastasis 3. Poor differentiation 4. Survival rate	–	[73, 74]
LAC	Tissues Cells		1. Cell proliferation	1. Tumor size	–	[75]
LAC	Tissues Cells		1. Cell proliferation 2. Cell apoptosis	1. Poor differentiation 2. TNM stage 3. Lymph node metastasis	–	[76]
NSCLC	Tissues Cells		1. Cell proliferation 2. Cell migration 3. Cell invasion 4. Cell apoptosis	1. Histological grade 2. N stage 3. TNM stage	–	[77]
NSCLC	Tissues Cells		1. Cell proliferation 2. Cell invasion	–	–	[80]
LAC	Tissues Cells		1. Cell proliferation 2. Cell apoptosis	1. TNM stage 2. Tumor size 3. T stage 4. Survival rate	Prognostic value	[42]
LAC	Tissues		–	1. T stage 2. Distant metastasis	Diagnostic values AUC: 0.815	[81]
NSCLC	Tissues Cells		1. Cell proliferation 2. Cell migration 3. Cell invasion 4. Cell apoptosis	1. TNM stage 2. Lymph node metastasis 3. Survival rate	Prognostic value	[82]
NSCLC	Tissues Cells		1. Cell proliferation 2. Cell migration 3. Cell invasion 4. Cell apoptosis	1. TNM stage 2. Lymph node metastasis 3. Survival rate	Prognostic value	[83]
NSCLC	Tissues Cells		1. Cell proliferation 2. Cell invasion	1. Tumor size 2. Lymph node metastasis 3. Survival rate	Prognostic value	[84]
NSCLC	Tissues Cells		1. Cell proliferation 2. Cell migration 3. Cell invasion 4. Cell apoptosis	1. Poor differentiation 2. Lymph node metastasis 3. T stage and N stage 4. Survival rate	Prognostic value	[85]
NSCLC	Tissues Cell		1. Cell proliferation 2. Cell apoptosis	–	–	[86]
NSCLC	Cells Mice		1. Cell proliferation 2. Cell migration	–	–	[87]
LAC	Tissues Cells Mice		1. Cell proliferation 2. Cell migration 3. Cell invasion 4. Cell apoptosis	4. TNM stage	–	[88]
NSCLC	Tissues cells		1. Cell proliferation 2. Cell invasion	1. TNM stage 2. Lymph node metastasis 3. Tumor size 4. Poor differentiation 5. Survival rate	Prognostic value	[89]
NSCLC	Tissues Cells Mice		1. Cell proliferation 2. Cell invasion	1. Survival rate	Prognostic value	[90]
NSCLC	Tissues Blood Cells		1. Cell proliferation 2. Cell migration 3. Cell invasion 4. Cell apoptosis	1. Distant metastasis	Diagnostic values Tissues: AUC: 0.803 Sensitivity: 0.825 Specificity: 0.675 Serum: AUC: 0.794 Sensitivity: 0.711 Specificity: 0.800	[91]
NSCLC	Tissues Cells		1. Cell proliferation 2. Cell apoptosis	1. Tumor size 2. TNM stage 3. Survival rate	Prognostic value	[92]
LAC	Tissues Cells		1. Cell proliferation 2. Cell apoptosis	1. TNM stage 2. Tumor size 3. Survival rate	Prognostic value	[93]
NSCLC	Tissues Cells		1. Cell proliferation 2. Cell invasion 3. Cell apoptosis	1. TNM stage 2. Lymph node metastasis	–	[94]
NSCLC	Tissues Cells		1. Cell proliferation 2. Cell invasion 3. Cell apoptosis	–	Diagnostic values AUC: 0.643 Sensitivity: 0.725 Specificity: 0.575	[95]
NSCLC	Tissues Cells		1. Cell proliferation 2. Cell migration 3. Cell invasion	1. TNM stage 2. Distant metastasis 3. Poor differentiation	–	[96]
NSCLC	Tissues Cells		1. Cell proliferation 2. Cell migration 3. Cell invasion 4. Cell apoptosis	1. Lymph node metastasis 2. Poor differentiation 3. Survival rate	–	[97]
NSCLC	Tissues Cells		1. Cell proliferation 2. Cell migration 3. Cell invasion 4. Cell apoptosis	1. TNM stage 2. Lymph node metastasis 3. Survival rate	Prognostic value	[98]
NSCLC	Tissues Cells		1. Cell proliferation 2. Cell invasion 3. Cell apoptosis	1. TNM stage 2. T stage 3. Poor differentiation 4. Survival rate	Prognostic value	[99]
NSCLC	Tissues Cells		1. Cell migration 2. Cell invasion	1. Lymph node metastasis 2. Prognostic value	Prognostic value	[100]
NSCLC	TISSUES Cells		1. Cell proliferation 2. Cell migration 3. Cell invasion 4. Cell apoptosis	–	–	[101]
NSCLC	Tissues Cells		1. Cell proliferation 2. Cell migration 3. Cell invasion 4. Cell apoptosis	1. TNM stage 2. Lymph node metastasis	Prognostic value	[102]
SCLC	Tissues Blood		1. Cell proliferation 2. Cell migration 3. Cell apoptosis	1. Lymph node metastasis 2. Survival rate	Prognostic value	[103]
NSCLC	Tissues Cells		1. Cell proliferation	1. Patients’ age (particularly after the age of 60) 2. TNM stage	–	[104]
NSCLC	Tissues Cells		1. Cell proliferation 2. Cell invasion 3. Cell apoptosis	1. TNM stage 2. Lymph node metastasis 3. Survival rate	Prognostic value	[105]
NSCLC	Tissues Cells		1. Cell invasion 2. Cell metastasis	–	–	[106]
NSCLC	Tissues Cells		1. Cell proliferation 2. Cell migration 3. Cell invasion	1. TNM stage 2. Lymph node metastasis 3. Survival rate	Prognostic value	[107]
NSCLC	Tissues Cells		1. Cell proliferation 2. Cell migration 3. Cell invasion	1. TNM stage 2. Lymph node metastasis 3. Survival rate	Prognostic value	[108]
NSCLC	Tissues Cells		1. Cell proliferation 3. Cell apoptosis	–	–	[109]
NSCLC	Tissues Cells		1. Cell proliferation 2. Cell migration 3. Cell invasion	–	Diagnostic values AUC: 0.782 Sensitivity: 0.800 Specificity: 0.733	[110]
LAC	Tissues Cells		1. Cell migration 2. Cell invasion	1. TNM stage 2. Lymph node metastasis	–	[111]
LAC	Tissues Cells		1. Cell proliferation 2. Cell migration 3. Cell invasion	1. TNM stage 2. Lymph node metastasis 3. Tumor size 4. Survival rate	Prognostic value	[112]
LAC	Tissues Cells		1. Cell proliferation 2. Cell migration 3. Cell invasion	1. T stage 2. Cell proliferation 3. Cell migration and invasion	–	[113]

CircRNAs are involved in the occurrence and progress of lung cancer, resulting in attracting considerable attention recently. Currently,Although a lot of literatures reported that circRNA can participate in the tumorigenesis and progress of lung cancer through various ways such as interaction with transcription factors and ceRNA mechanism, ceRNA mechanism is still the main focus among diverse circRNA functions in the tumorigenesis of lung cancer. In this review, we list all existing studies on circRNA-miRNA-mRNA axis in lung cancer, throwing light on further research on circRNAs in lung cancer, and even giving new ideas for early diagnosis and precise treatment of lung cancer.

It is reported that some circRNAs interacting with miRNA are overexpressed in lung cancer. Qu et al. found that hsa_circ_0020123, identified as the sponge of miR-144, is upregulated in NSCLC, thus leading to uprising of ZEB1/EZH2. The phenomenon that this regulatory axis may associate with the tumor differentiation, lymph node metastasis, TNM stage and survival rate in NSCLC can be found in the statistics data of a large collection of human cancer tissues. Simultaneously, hsa_circ_0020123 also has potential to give an easy access to early-diagnosis for NSCLC [85]. Almost all the researches about lung cancer focus on NSCLC, while only one literature about SCLC can be found in the database described above. Li et al. identified that circFLI1, as an ecircRNA, is involved in the occurrence of metastasis, which is dramatically up-regulated in the SCLC tissues compared with NSCLC tissues. Of interest, there are no significance between circFLI1 and cell proliferation, apoptosis and colony formation. They also found the regulatory network in circFLI1s that circFLI1 binds to miR-584-3p which further regulating its downstream targets-RhoA/ROCK1 signal pathway [103].

On the other hand, expression of some circRNAs in lung cancer significantly lower than in normal tissues or para-carcinoma tissues. Yang et al. reported that hsa_circ_0046264 is associated with lymph node metastasis and TNM stage in NSCLC, and it plays a vital role through hsa_circ_0046264↓-miR-1245↑- BRCA2↓ axis [105]. Moreover, Chen et al. found that low expression of hsa_circ_100395 in NSCLC can affect the TNM stage, metastasis and survival rate in NSCLC. Further study explores that hsa_circ_100395 modulating miR-1228 regulates lung cancer cells proliferation, migration and invasion. The hypothesis derived from RNA-seq profile that hsa_circ_100395 preforms its function by targeting miR-1228/TCF21 axis can be confirmed by luciferase reporter assay and overexpression experiment [107].

According to WHO cancer report, lung cancer has become the first killer among all cancers, leading to one out of five deaths of cancer-related mortality [114]. Although the chemotherapy is still available, it is noteworthy that once patients develop to chemotherapy resistance, the survival rate will be dramatically decreased, leading to higher mortality in the population of suffering lung cancer. It is reported that resistance of canonical chemotherapy like cisplatin and targeted therapy like TKI have occurred in clinical setting, which become the barriers to treatment [115, 116]. Strikingly, Zhou et al. shown that upregulation of hsa_circ_0004015 can remarkably increase the resistance of gefitinib in resistant cancer cells HCC827I/R, while downregulation can dramatically decrease the gefitinib IC50 of resistant cancer cells. Likewise, they also found that hsa_circ_0004015 functions through miR-1183/PDPK1 regulatory axis, which selectively promotes progression and invasion of lung cancer, but no significance can be found in the relationship between hsa_circ_0004015 and metastasis, no matter lymphatic metastasis or distant metastasis [99]. Therefore. the ceRNA mechanism of circRNA may play an indispensable role in chemotherapy resistance or susceptibility, which provides a brand-new perspective for this research.

After summarizing the literatures of circRNA-miRNA-mRNA regulatory network in lung cancer, we can clearly see that there are some interactions between different circRNAs regulatory axes. We describe this phenomenon as follow in terms of two parts.

### Interactions of miRNAs in different circRNA-miRNA-mRNA network

*miR-7* There are some different opinions about the relationship between miR-7 and lung cancer oncogenesis. Canonically, ciR-7 identified as a sponge of miR-7 acts on downstream targets such as NF-κB, EGRF, CCNE1, PIK3CD, eventually contributing to cell proliferation, migration and metastasis [71, 72]. Acting like an oncogene, miR-7 is downregulated in NSCLC tissues and also inhibits the cell proliferation, migration and invasion by suppressing ERK/MAPK signaling pathway [117]. Strikingly, however, Wan et al. reported that miR-7 sponged by circICTH enhances lung cancer cell proliferation. They also found that miR-7 can degrade the expression level of circITCH. Moreover, circITCH competitively inhibited miR-7 binding to ITCH remarkably enhances the expression of ITCH which promotes the oncogenesis of lung cancer by activating Wnt/β-catenin signal pathway [104].

*miR-326* It is reported that that both hsa_circ_0003998 and circPUM1 are able to function as a sponge of miR-326. Differentially, hsa_circ_0003998 as a sponge of miR-326 modulate the downstream Notch1, while circPUM1 binding to miR-326 regulates targeted genes, cyclin D1 and Bcl-2 [84, 88]. Either hsa_circ_0003998 or circPUM1 considerably increases in lung cancer tissues and cells and contributes the tumor-promoting effect by different signal pathways. What’s more, Hang et al. also reported that circFARSA is upregulated in lung cancer and has predicted binding sites of miR-326. Experimentation of RPI and dual-luciferase reporter assay are needed to further confirm the relationship between cicrFARSA and miR-326.

*miR-1179* miR-1179 sponged by both hsa_circ_0000735 and hsa_ circ_0003645 is downregulated in NSCLC tissues and cells [94, 98]. An et al. shown that circ_0003645 facilitates the promotion of cancer cell growth, apoptosis, and metastasis through miR-1179/TMEM14A regulatory axis [98].

### Interactions of mRNAs in different circRNA-miRNA-mRNA network

Wnt/β-catenin signaling pathway: As is described in the above table, there are four circRNA regulatory networks which involved in the common downstream targeted pathway, Wnt/β-catenin signaling pathway. has_circ_0001946, has_circ_001569, has_circ_0006427 and circITCH will be drawn our attention due to their sharing signal pathway. has_circ_0001946 sponges miR-135a-5p and activates Wnt/β-catenin pathway by enhancing Sirtuin 1 (SIRT1) protein [93]. It is known that SIRT1 contributes its effect of tumor promotion to tumorigenesis of lung cancer. Recently, Lee et al. reported that Metformin and tenovin‐6 targeting downregulated SIRT1 synergistically induce apoptosis in lung cancer cell [118]. has_circ_001569 preforms its function by miRNA-148a, enhancing Wnt/β-catenin pathway associated gene expression [119]. has_circ_0006427 identified as miR-6783-3p sponge was found to be downregulated in NSCLC. The finding that, differently from has_circ_0001946, has_circ_0006427 activates its downstream pathway by increasing DKK1 protein [112]. Likewise, circITCH is also dramatically downregulated in NSCLC with binding sites of miR-7 and miR-214. Overexpression of circITCH can remarkably inhibits the activation of Wnt/β-catenin signal pathway through declining the vital downstream gene, c-Myc and cyclinD1 [104]. Shortly, although these four circRNAs share the common important signal pathway that significantly promote lung cancer cell in terms of growth, invasion and metastasis, they activate the pathway by targeting different proteins in the cell.

*Cyclin D1 and bcl-2* Zhu et al. reported that hsa_circ_0013958 can promote LAC by regulating cyclin D1, while Chen et al. found that circPUM1 can also perform its function by targeting cyclin D1 and bal-2 [69, 88]. Additionally, hsa_circ_0000064 is reported to upregulate in NSCLC by modulating cyclin D1, bcl-2 and so on which is not described in our table [120]. Besides circPUM1 and hsa_circ_0000064, hsa_circ_0007385 and circPVT1 can also respectively bind to miR-181 and miR-497 and target the same downstream gene, bcl-2 [78, 91].

Briefly, both Wnt/β-catenin and Cyclin D1, bcl-2 are the common signal pathway affecting cell proliferation, apoptosis, invasion and migration in lung cancer. Some circRNAs binding to different miRNAs share the same downstream targets. In the era of precision medicine, blocking these common pathways maybe is a good idea to inhibit the growth and prevent the metastasis in lung cancer.

## Clinical characteristics of circRNAs in lung cancer

**Fig. 1:**
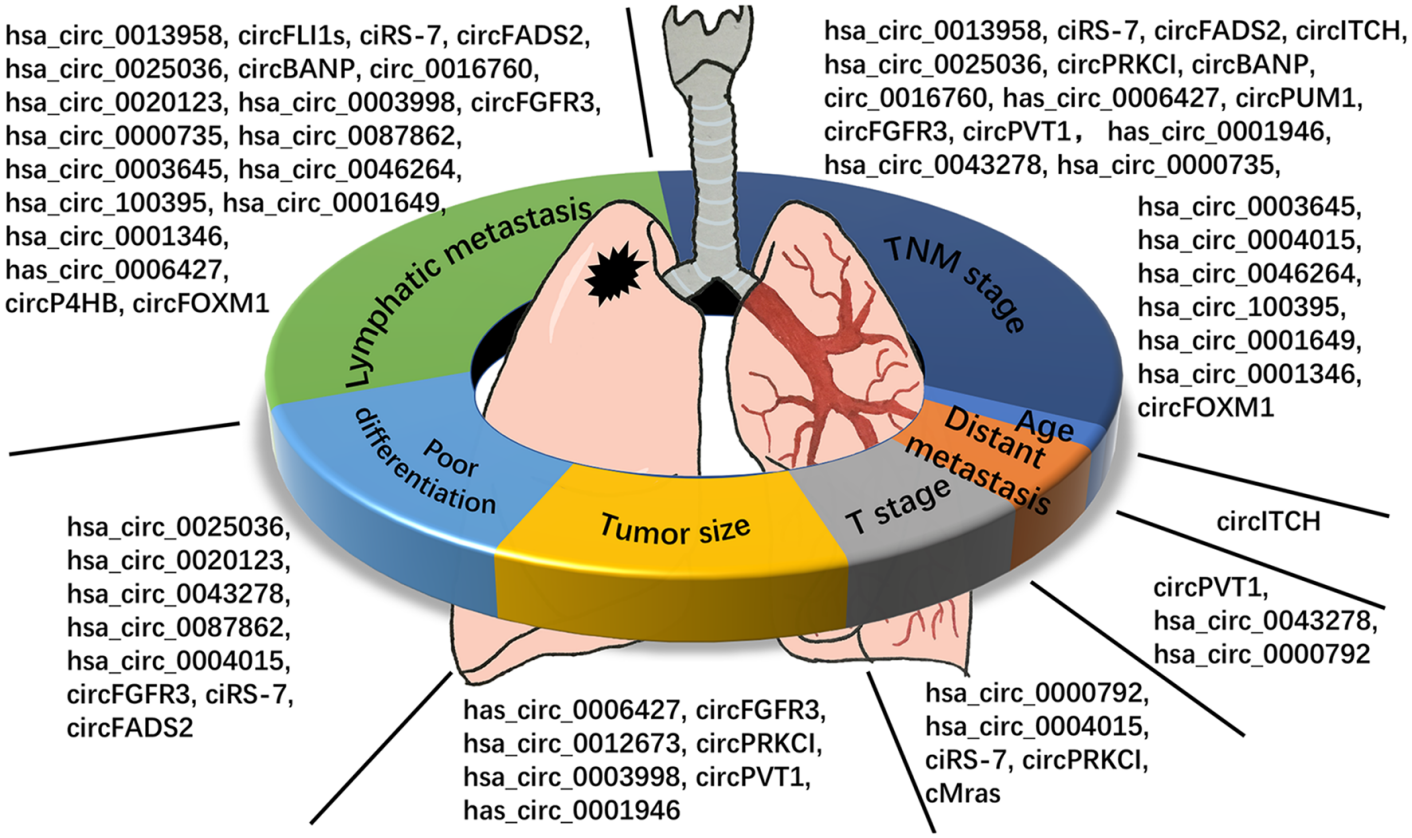
The summary of clinical functions in lung cancer. All the circRNAs were concluded from validated circRNAs with circRNA-miRNA-mRNA regulatory network. CircRNAs included in our study were divided into 7 parts according to their clinical characteristics. As we can see in the picture, most of the circRNAs are significantly related to lymphatic metastasis and TNM stage

**Fig. 2:**
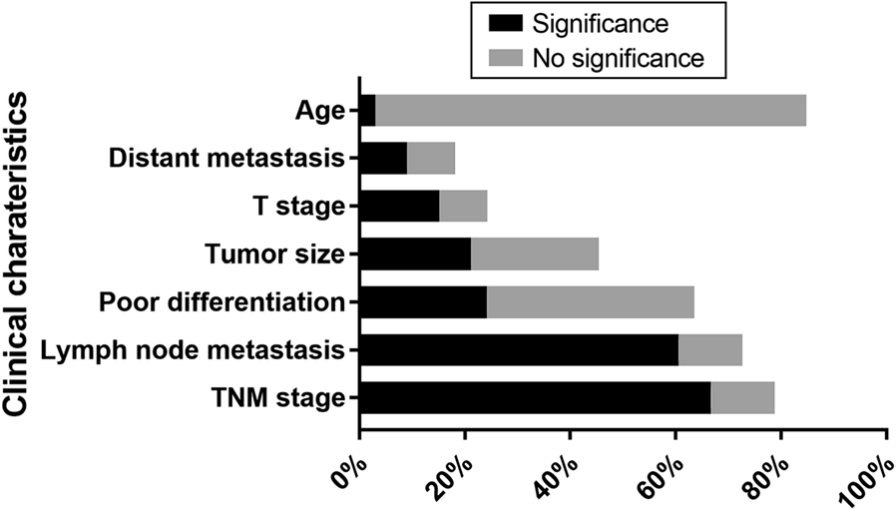
Summary of clinical characteristics of circRNAs in lung cancer. Percent in X axis means the studies involved in each clinical event take up in the whole studies about circRNA-miRNA-mRNA regulatory axis in lung cancer. Significance in each group means there is a significant relationship between clinical characteristics and certain circRNA. No significance in each group means no significance can be found between clinical characteristics and certain circRNA

As we can see in the Table 2, circRNAs have multiple clinical characteristics in lung cancer. We summarize the clinical characteristics of circRNAs with ceRNA-miRNA-mRNA regulatory function in lung cancer (Figs. 1 and 2). It is clear that the most widely studied characteristics of circRNAs in lung cancer are TNM stage and lymphatic metastasis, both of which take up nearly 80% of all availably found studies. Meaningfully, we can find that there is a close relationship between circRNAs and TNM stage and lymphatic metastasis in lung cancer. We are delighted that a large amount of circRNAs have potential to develop into excellent biomarkers of lymphatic metastasis in lung cancer. Both distant metastasis and T stage are the characteristics which absorb least attention to researchers. Suggestion can be taken from these phenomena that maybe more circRNAs are significantly involved in distant metastasis and T stage in lung cancer if researchers play more attention to distant metastasis and T stage. Statistics of patients’ age can be found in almost all the circRNA studies of lung cancer, but only one circRNA is significantly related to patients’ age. Hence, if we can make good use of the significant relationship between circRNAs and TNM stage, lymphatic metastasis in lung cancer, maybe circRNA can be developed into an excellent prognostic biomarker of clinical characteristics.

## Conclusion and perspective

**Fig. 3:**
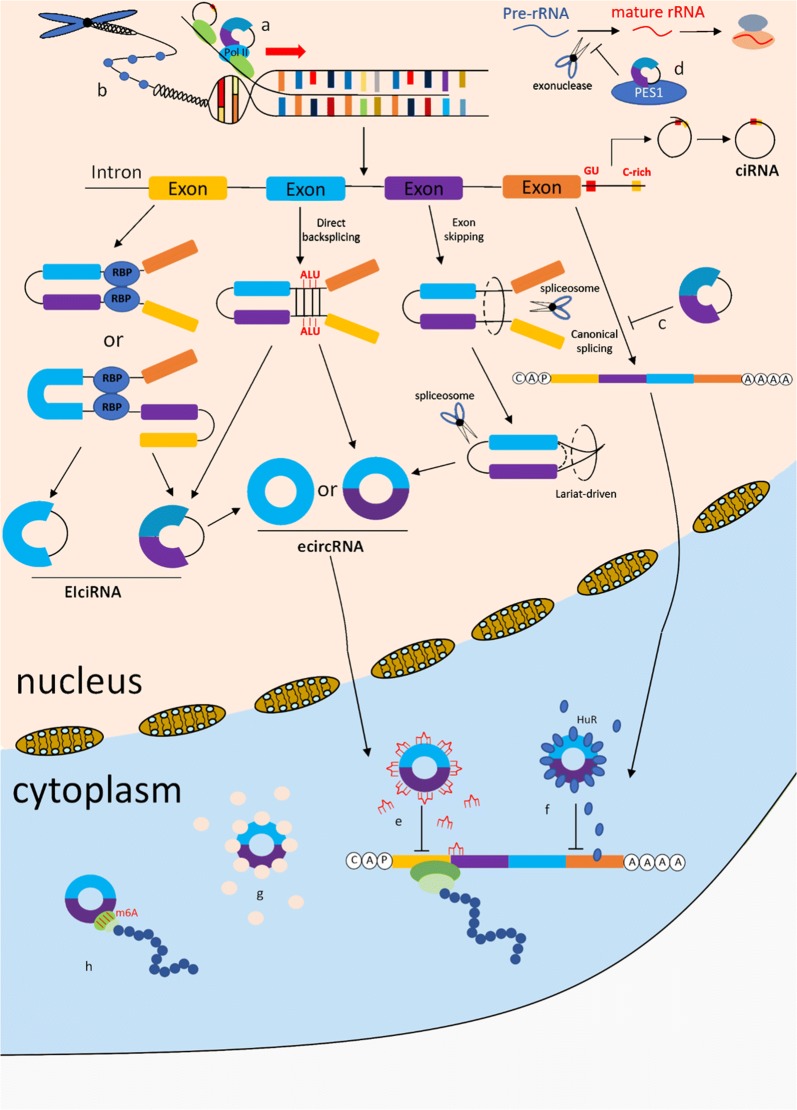
The whole view of the biogenesis and function of circRNAs. The biogenesis of three kinds of circRNA (EIciRNA, ecircRNA and ciRNA) and eight kinds of function, including circRNA-miRNA-mRNA axis, were presented in the picture. **a** RNAP II elongation. **b** Histone methylation. **c** Alternative splicing. **d** RNA maturation. **e** miRNA sponge. **f** Translation splicing. **g** Protein sponge. **h** Protein translation (including m6A-driven)

The summary of biogenesis and function characteristics of circRNAs shows in Fig. 3. In recent years, numerous of circRNAs have been found and investigated. circRNAs affect the expression of related genes by acting as miRNA sponges. Additionally, circRNAs are abundant and stable in cells, blood, tissues, exosomes and saliva, providing a new way of understanding the occurrence and development of diseases.

Despite the discovery of thousands of circRNAs, our understanding of circRNAs is still limited, especially in terms of their biogenesis and functions. Moreover, although the circRNAs are known as a new kinds of potential cancer biological markers and therapeutic targets, there still exist many problems in the clinical application of circRNAs, such as high medical expense, complex monitoring approach, and so on. Therefore, we need universal study to shed light on the development and clinical application of circRNAs so that it may provide new ideas for early diagnosis and treatment of lung cancer.

## Data Availability

Not applicable.

## Abbreviations

ncRNA: Non-coding RNA
miRNA: MicroRNA
lncRNA: Long non-coding RNA
circRNA: CIRCULAR RNA
RNA-seq: RNA sequencing
mRNA: Message RNA
pre-mRNA: Precursor mRNA
ecircRNA: Exonic circRNA
EIciRNA: Exonic-intronic circRNA
ciRNA: intronic circRNA
tricRNA: tRNA intronic circRNA
circrRNA: CircRNA rRNA
CS: Constitutive splicing
AS: Alternative splicing
3′ ss: 3′ upstream splice site
5′ ss: 5′ downstream splice site
RBP: RNA binding protein
MBL: Muscleblind
QKI: Quaking
FUS: Fused-in sarcoma
HCC: Hepatocellular carcinoma
EMT: Epithelial-to-mesenchymal transition
ORF: Open reading frame
m6A: N6-methyladenosine
PES1: Pescadillo homologue 1
ceRNA: Competing endogenous RNA
MRE: miRNA response element
ESCC: Esophageal squamous cell carcinoma
TNBC: Triple negative breast cancer
MMP: Matrix metalloproteinase
SIRT1: Sirtuin 1
TME: Tumor microenvironment
PD-1/PD-L1: Programmed death 1/programmed death-ligand 1
NK: Natural killer
GEC: Glioma associated endothelial cell
